# Case Report: Primary gallbladder undifferentiated small round cell sarcomas treated with gemcitabine, paclitaxel, bevacizumab and pembrolizumab achieved remarkable tumor regression

**DOI:** 10.3389/fimmu.2026.1719285

**Published:** 2026-03-17

**Authors:** Dan Liu, Xiaoge Liu, Mou Li, Xin Li

**Affiliations:** 1 Department of Radiology, Sichuan Provincial People’s Hospital, University of Electronic Science and Technology, Chengdu, Sichuan, China; 2 Department of Ultrasound, Ya’an People’s Hospital, Ya an, Sichuan, China

**Keywords:** undifferentiated small round cell sarcoma, Ewing sarcoma, gemcitabine, programmed death-1 inhibitor, vascular endothelial growth factor receptor

## Abstract

Undifferentiated small round cell sarcoma (USRCS) are a rare, aggressive group of tumors associated with rapid progression, metastasis, and poor prognosis. Preoperative diagnosis remains elusive, particularly for extraosseous variants, and effective treatments are lacking. This case reports the use of pembrolizumab, bevacizumab, and conventional chemotherapy (gemcitabine plus paclitaxel) as a first-line treatment for a patient with primary USRCS in the gallbladder neck; the patient achieved remarkable partial remission for more than 9 months. This case represents the second documented instance of gallbladder USRCS and the first managed with this specific combination regimen. These findings suggest that integrating immunotherapy and targeted agents with conventional chemotherapy may offer a promising therapeutic strategy for USRCS.

## Introduction

Undifferentiated small round cell sarcomas (USRCS) constitute a rare and aggressive group of neoplasms, predominantly affecting the bones and soft tissues of children and young adults (1). While Ewing sarcoma (ES) remains the prototypic entity, the World Health Organization (WHO) classification now recognizes four distinct USRCS subtypes: ES, CIC-rearranged sarcomas, round cell sarcomas with *EWSR1-non-ETS* fusions (e.g., *NFATC2, PATZ1*), and sarcomas with *BCOR* genetic alterations (2). Although these tumors typically arise in the skeletal system or deep soft tissues of the trunk and extremities, extraosseous manifestations have been documented in visceral organs, including the gastrointestinal tract, kidney, and adrenal gland (3). Gallbladder involvement is exceedingly rare; to our knowledge, only one case of a primary primitive neuroectodermal tumor (PNET) has been reported by Song et al. (4), along with a single instance of synchronous ES/PNET involving the gallbladder reported by Plis et al. (5).

Current management strategies for USRCS are largely extrapolated from ES protocols, involving multimodal therapy with neoadjuvant chemotherapy followed by local treatment (6). Standard regimens typically include vincristine, doxorubicin, and cyclophosphamide (VAC), or ifosfamide and etoposide (IE). More recently, the gemcitabine and docetaxel (GD) combination has shown efficacy against advanced ES (7, 8), with studies exploring the addition of bevacizumab to enhance outcomes (9). Furthermore, emerging evidence supports the potential of immune checkpoint inhibitors (ICIs) in ES (10). Herein, we report the first case of primary gallbladder USRCS treated with a novel combination of gemcitabine, paclitaxel, bevacizumab, and pembrolizumab as first-line therapy. The patient achieved durable disease control exceeding nine months with good tolerance and no severe adverse events. This case suggests that integrating immunotherapy and targeted agents with chemotherapy may represent a promising therapeutic strategy for this aggressive malignancy.

## Case presentation

**Figure 1:**
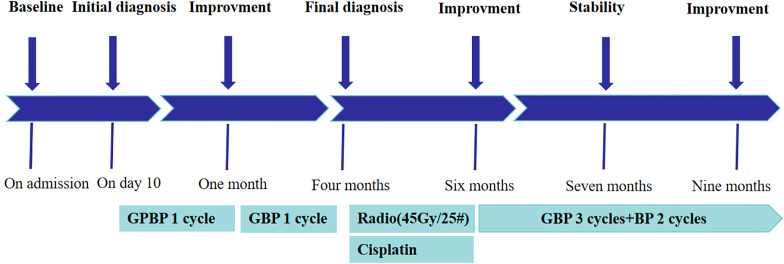
Time points were calculated from the date of admission. GBPB, gemcitabine, paclitaxel micelle, beacizumab, pembrolizumab; GBP, gemcitabine, beacizumab, pembrolizumab.

**Figure 2:**
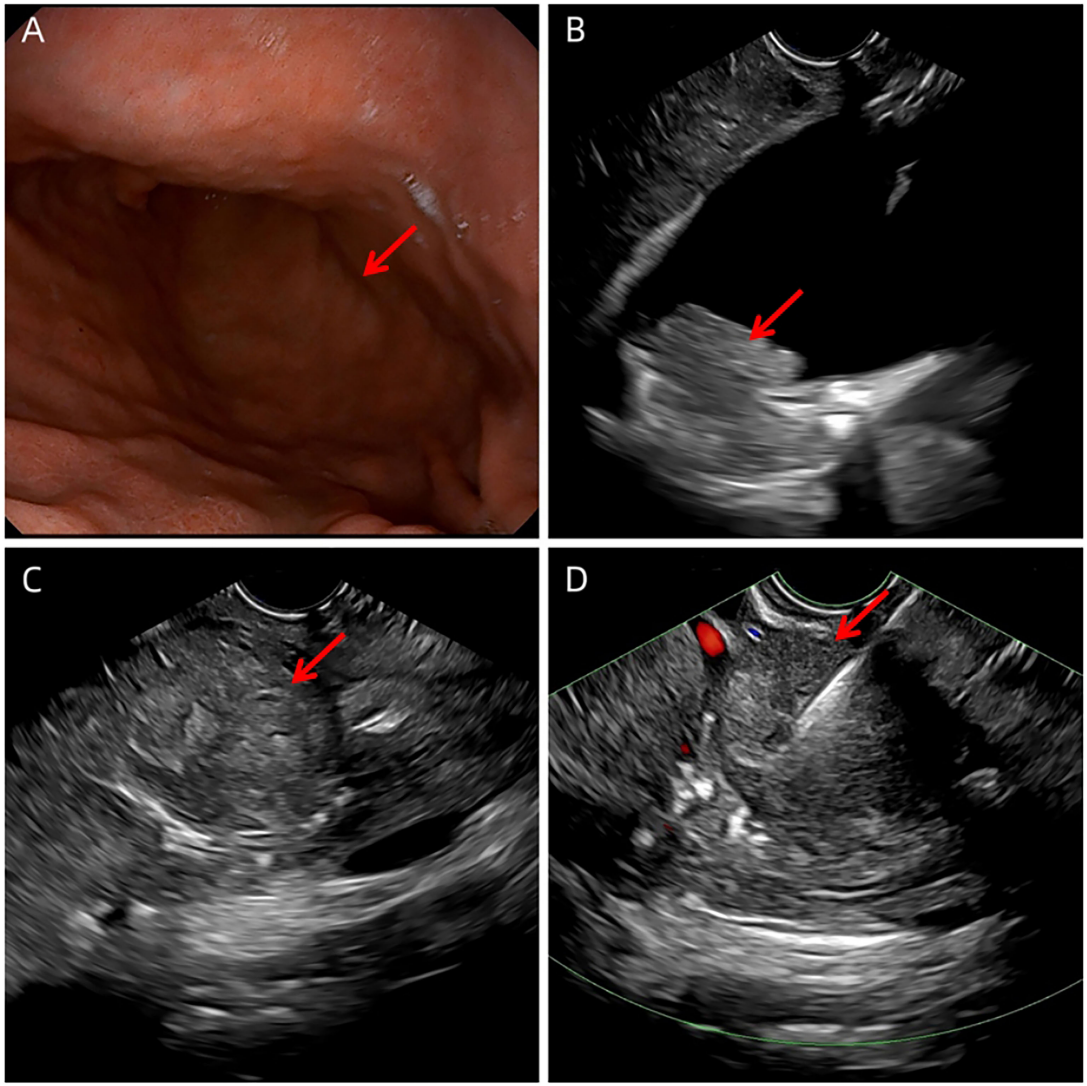
Radiological and endoscopic evaluation of the patient. **(A)** Endoscopic image showing a submucosal protrusion in the stomach, caused by an enlarged retroperitoneal lymph node protruding into the gastric lumen. **(B)** Abdominal ultrasound image demonstrating a solid, dumbbell-shaped mass located at the neck of the gallbladder (arrows),indicating invasion through the gallbladder wall. **(C)** Ultrasound image showing the enlarged retroperitoneal lymph node. **(D)** Ultrasound image guiding fine-needle aspiration (FNA).

**Figure 3:**
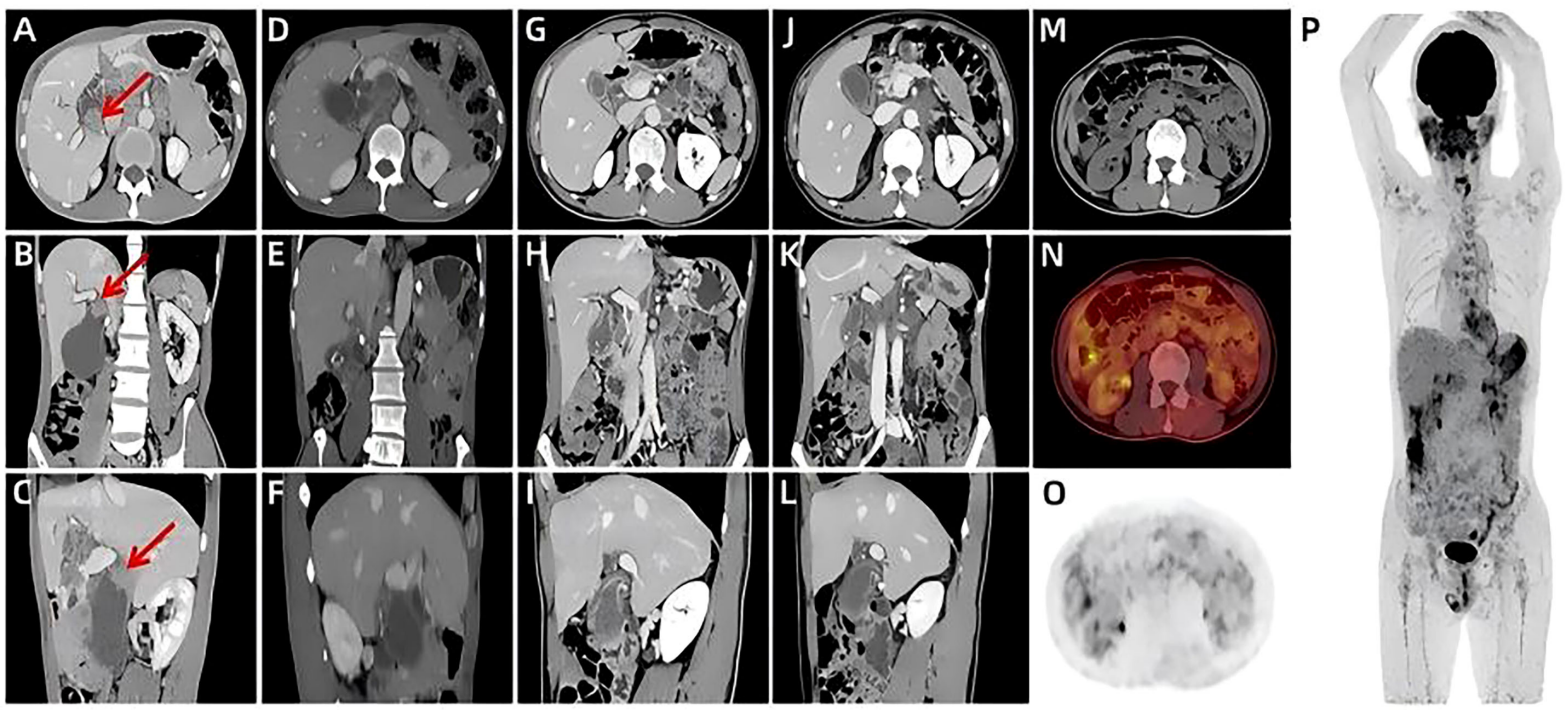
Abdomen computed tomography images: **(A–C)** Baseline before treatment (red arrows),contrast-enhanced CT scans at admission revealed a mass in the gallbladder neck and enlarged lymph nodes; after treatment, follow-up CT scans at one **(D–F)**, three **(G–I)** and six months **(J–L)** post-admission, along with a PET-CT at seven months **(M–P)**, demonstrated complete resolution of the primary lesion and reduction of lymphadenopathy.

**Figure 4:**
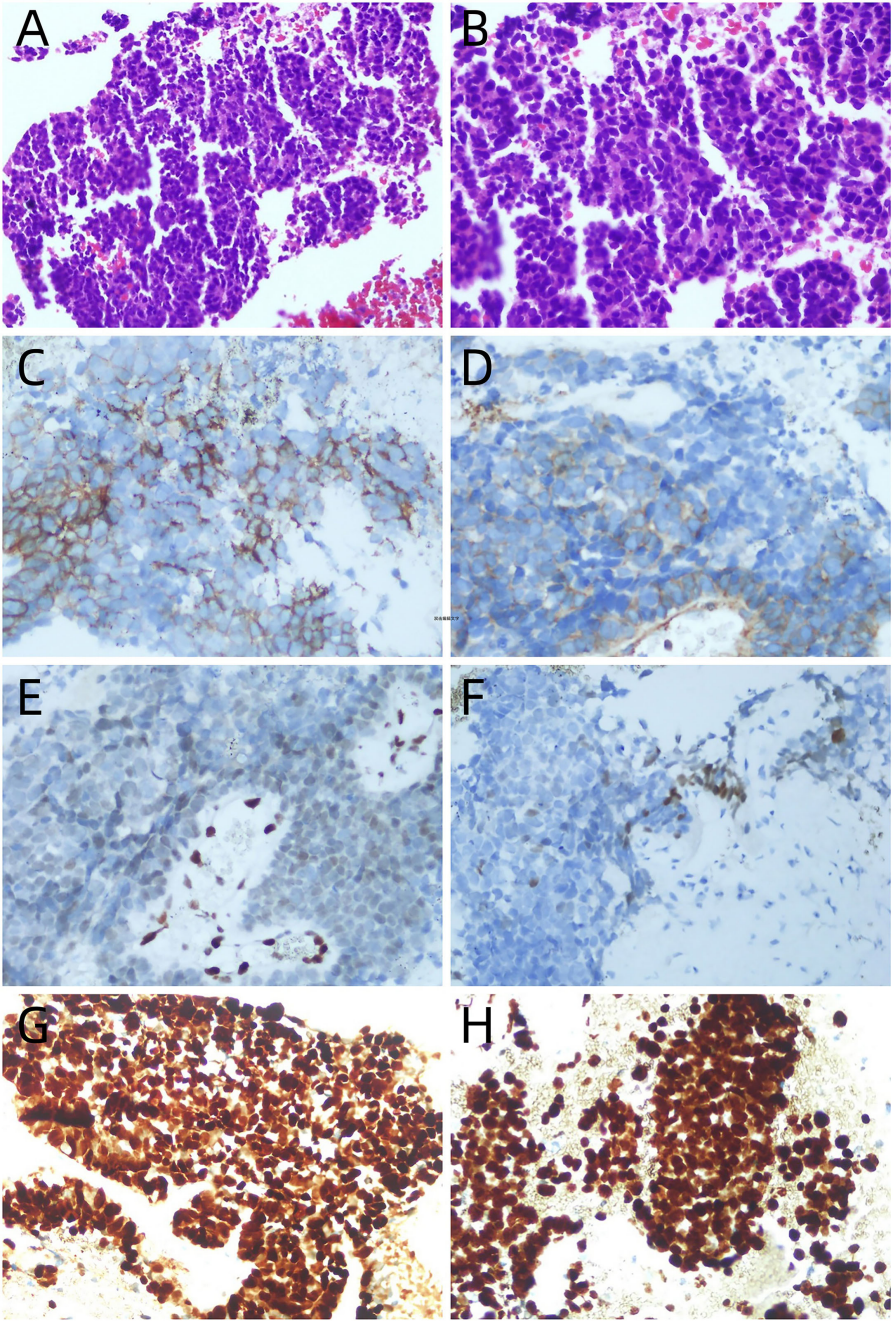
**(A)** (H&E ×200) Atypical small round blue cells with diffuse growth pattern without hyalinized fibrous tissue. **(B)** (H&E ×400) The tumor cells were small round with sparse cytoplasm, round nuclei, finely dispersed chromatin and small inconspicuous nucleoli, suggesting small round cell undifferentiated sarcoma. **(C–H)** Immunohistochemical result of this case: **(C)** CD56 (positive, diffuse); **(D)** CD99 (positive, diffuse); **(E)** Fli-1 (positive, diffuse); **(F)** NKX2.2 (positive, diffuse); **(G)** CyclinD1 (positive, diffuse); **(H)** the proliferative activity (Ki-67) index was about 90%.

The clinical timeline is shown in **Figure 1**. A 60-year-old male presented with a one-month history of epigastric pain, abdominal distention, and nausea. Six days prior to admission, abdominal computed tomography (CT) scan at an outside hospital revealed a polypoid, heterogeneously enhancing mass in the gallbladder, accompanied by numerous enlarged lymph nodes. Then he was referred to our hospital with a suspected diagnosis of gallbladder cancer. The patient had no remarkable past medical history. Physical examination revealed a non-palpable gallbladder and a negative Murphy’s sign. Laboratory tests, including a complete blood count and biochemical parameters, were within normal limits. On day 2 of admission, ultrasonography identified a solid nodule (1.7×2.3cm) in the gallbladder neck and confirmed the presence of widespread lymphadenopathy (**Figure 2**). An enhanced CT scan performed concurrently demonstrated an irregular soft-tissue mass in the gallbladder neck (approximately 1.9×2.2×2.9cm) with heterogeneous enhancement and extension through the serosa. Multiple enlarged lymph nodes were observed in the peritoneal and retroperitoneal spaces, with the largest lesion measuring 2.9×3.0×3.5 cm. These nodes caused significant compression of major vasculature, including the portal vein, splenic vein, inferior vena cava, celiac axis, superior mesenteric artery, and pancreatic head but without definite evidence of invasion. Additionally, multiple gallstones were noted (**Figure 3**). Based on these findings, the disease was deemed unresectable. Cytologic examination of ascitic fluid revealed clusters of cells with pleomorphic nuclei. To obtain a definitive diagnosis, the patient underwent endoscopic ultrasound-guided needle biopsy of an abdominal lymph node. Preliminary pathology suggested an atypical small round cell tumor. On day 10, initial immunohistochemistry (IHC) showed positivity for Cyclin D1, Syn, and CD10 and negativity for BCL2, BCL6, CD3, CD20, CD21, CDX2, CgA, INSM1, and MUM1. The Ki-67 index was 90%. ES was suspected. Consequently, a second round of IHC and fluorescence *in situ* hybridization (FISH) was scheduled. After excluding contraindications, the clinicians initiated empiric chemotherapy with a GPBP regimen (gemcitabine 1 g, paclitaxel micelle 300 mg, bevacizumab 600 mg, and pembrolizumab 200 mg on days 1 and 8 of a 3-week cycle) on day 15 of admission, pending a definitive diagnosis. On day 16, the second round of IHC results (**Figure 4**) revealed strong positivity for CD56, CD99, NKX2.2, Fli1, CK, and p63, and negativity for S-100, DES, ERG, WT1, and Cam5.2. Nine days after initiating chemotherapy, a planning CT scan showed a reduction in the gallbladder lesion and abdominal lymph nodes (**Figure 3**). Given the patient’s improved condition but severe alopecia, paclitaxel micelle was discontinued. The treatment plan was modified to continue with a GBP regimen (gemcitabine, bevacizumab, and pembrolizumab), followed by two cycles of cisplatin-based chemotherapy and stereotactic body radiation therapy (45 Gy/25 fractions). Two months after admission, FISH analysis was negative for *EWSR1* gene rearrangement. Due to technical limitations, further molecular subtyping could not be performed. Based on the constellation of cytological, histological, and IHC findings, a diagnosis of primary gallbladder USRCS was established. Whole-body CT staging confirmed the absence of distant metastases. Given the patient’s clinical improvement and positive radiological response, the GBP chemotherapy regimen was continued for three additional cycles. Whole-body CT scans at three and six months post-admission, followed by a PET-CT seven months after admission, consistently demonstrated complete resolution of the gallbladder lesion with a reduction in abdominal lymphadenopathy (**Figure 3**). The patient then received two cycles of maintenance immunotherapy and targeted therapy, which were well-tolerated without any immune checkpoint inhibitor-related toxicities. The patient achieved a progression-free survival (PFS) of 9 months; however, follow-up CT performed at 11 months post-admission revealed disease progression, characterized by enlarging retroperitoneal lymph nodes and new hepatic and pancreatic metastases. The patient ultimately succumbed to the disease, with an overall survival (OS) of 13 months.

## Discussion

Undifferentiated small round cell sarcoma (USRCS) of the gallbladder is exceedingly rare, presenting significant diagnostic and therapeutic challenges. In this case, preoperative imaging suggested a primary gallbladder malignancy with lymph node metastasis. Although definitive IHC and FISH analysis confirmed the diagnosis of gallbladder USRCS, the patient had already been initiated on a gallbladder cancer-specific chemotherapy regimen. Notably, this regimen resulted in a significant reduction of lesions and led to a period of remission, suggesting the potential efficacy of gallbladder cancer protocols for this rare entity.

USRCS represents a heterogeneous group of rare tumors. Classic Ewing sarcoma (ES) is by far the most common member of this group, characterized by monomorphic small round cell proliferation, strong and diffuse membranous CD99 expression, and *EWSR1/FUS::ETS* fusions. In contrast, the presence of pleomorphism, fibro-hyalinized or myxoid stroma, spindle, epithelioid, or rhabdoid cells, or negative/patchy CD99 expression should prompt consideration of alternative diagnoses. These include round cell sarcomas with *EWSR1-non-ETS* fusions, CIC-rearranged sarcomas, and BCOR-altered sarcomas. Consequently, a comprehensive approach integrating histology, IHC, and molecular evaluation is essential for accurate subtyping. Although next-generation sequencing (NGS), FISH, and reverse transcription polymerase chain reaction (RT-PCR) represent the gold standards for definitive diagnosis, their implementation remains challenging. Molecular testing is often limited by resource constraints or insufficient biopsy material, particularly in low- and middle-income countries (11). These barriers frequently lead to diagnostic delays and may result in patients initially receiving treatment regimens designed for morphological mimics, which often prove inadequate once the correct molecular diagnosis is established.

Due to the rarity of USRCS, optimal management remains undefined, and treatment typically follows protocols established for ES (12). Standard ES treatment involves a multimodal approach combining systemic and local therapies, which has achieved 5-year survival rates of 70-80% for localized tumors (13). Local control, usually involving surgery and/or radiotherapy, is generally deferred until after the initial chemotherapy (14). Traditional regimens often comprise vincristine, dactinomycin, cyclophosphamide, doxorubicin, ifosfamide, and etoposide (15). However, our patient was initially misdiagnosed with gallbladder cancer and received standard gallbladder cancer therapy. Remarkably, the first cycle of chemotherapy induced a significant reduction in lesion size on CT. Despite subsequent IHC and FISH analyses confirming USRCS, the patient continued on the non-standard regimen (gemcitabine, bevacizumab, and pembrolizumab), supplemented by cisplatin and radiotherapy. This approach yielded favorable clinical outcomes.

Biliary tract cancers (BTCs) encompass cholangiocarcinoma (intrahepatic, perihilar, and extrahepatic) and gallbladder cancer (GBC). GBC, the most common and aggressive malignancy within this group, carries a poor prognosis. It is frequently diagnosed at an advanced stage, often incidentally during cholecystectomy for presumed benign gallstone disease (16). For advanced BTCs, the standard first-line chemotherapy is the combination of gemcitabine and cisplatin (GC) (17). Currently, the National Comprehensive Cancer Network (NCCN) guidelines recommend GC plus durvalumab, an inhibitor of programmed cell death ligand 1 (PD-L1), as the preferred regimen for unresectable BTCs (18). Furthermore, chemotherapy regimens integrated with bevacizumab have shown promising efficacy in the treatment of BTCs (19).

While gemcitabine combined with cisplatin constitutes the standard first-line regimen for gallbladder cancer, gemcitabine has also been investigated in combination with other agents for ES (8, 20); however, optimal therapeutic combinations for ES remain under investigation. The GEIS-21 trial, a prospective multicenter study by the Spanish Sarcoma Group, evaluated the efficacy of gemcitabine and docetaxel (G/D) as a window-phase therapy in patients with high-risk ES. The study reported an objective response rate of 70% (12/17), suggesting significant clinical benefit for newly diagnosed high-risk patients (8). Conversely, Fox et al. (21) explored gemcitabine plus nab-paclitaxel in recurrent ES, observing an objective response rate of 9%. This outcome is comparable to response rates documented for G/D in multi-institutional studies involving similar patient populations.

Bevacizumab is a humanized monoclonal antibody that inhibits angiogenesis by blocking the interaction between vascular endothelial growth factor (VEGF) and its receptors. As a key proangiogenic factor, VEGF drives endothelial cell mitogenesis and plays a critical role in tumor growth and metastasis (22). Consequently, angiogenesis is a recognized therapeutic target in pediatric solid tumors, given the association between angiogenic factor expression and poor prognosis (23). Preclinical studies have validated the potential of VEGF pathway inhibition in ES (24) and neuroblastoma (25). Clinically, Wagner et al. (26) reported responses in extraosseous ES patients treated with vincristine, irinotecan, temozolomide, and bevacizumab. Similarly, Kuo et al. (9) demonstrated a 67% objective response rate using docetaxel, bevacizumab, and gemcitabine in ES patients. Consistent with these findings, our patient achieved a remarkable therapeutic response following maintenance chemotherapy with gemcitabine and bevacizumab, resulting in the resolution of the primary tumor and reduction of abdominal lymph nodes on follow-up CT.

Pembrolizumab, a humanized IgG4 kappa anti-PD-1 monoclonal antibody, inhibits the interaction between the PD-1 receptor and its ligands, PD-L1 and PD-L2. It has demonstrated efficacy across various malignancies, including advanced melanoma, non-small cell lung cancer, and Hodgkin lymphoma (27, 28). In sarcomas, PD-L1 expression varies significantly, reported in 57% of ES cases and reaching up to 86% in alveolar rhabdomyosarcoma. The high prevalence of PD-L1 in ES, combined with the genomic instability characteristic of this disease, provides a strong rationale for using immune checkpoint inhibitors (29, 30). Supporting this, McCaughan et al. (10) reported the first successful case of PD-1 blockade in ES, and Scheinberg et al. (31) subsequently described durable responses to pembrolizumab in a subset of adolescent and young adult patients. These findings suggest that immunotherapy may be a viable option for specific ES patients. Furthermore, early-phase clinical trials are currently evaluating diverse immunotherapeutic strategies—including checkpoint inhibitors, antibody-based therapies, and T-cell therapies—for patients with USRCS (32).

The management of primary gallbladder small round cell tumors has evolved significantly. Song et al. (4) reported a gallbladder PNET treated surgically without adjuvant therapy. While this achieved short-term local control for early-stage disease, it potentially increased the risk of recurrence given the aggressive nature and high relapse rates (30–40%) associated with the ES/PNET family (33). In contrast, the present case of unresectable gallbladder USRCS necessitated a multimodal approach. The patient received empiric chemotherapy (GPBP) incorporating immunotherapy and targeted agents, followed by cisplatin and stereotactic body radiation therapy (SBRT), which achieved an initial complete radiological response. Ultimately, however, the patient succumbed to the disease 13 months post-diagnosis. This comparison highlights that while surgery remains the standard for resectable tumors, the integration of novel systemic and local therapies is essential for managing unresectable, aggressive variants. The current case exemplifies the modern, multimodal strategy necessary for advanced, unresectable disease. Future management of these rare tumors will likely require a balance between aggressive surgical intervention when possible and the integration of novel systemic therapies (immunotherapy, targeted therapy) to improve long-term survival outcomes.

## Conclusion

Undifferentiated small round cell sarcomas (USRCSs) pose significant diagnostic and therapeutic challenges due to their rarity, atypical locations, and overlapping histopathological features. Primary gallbladder USRCS is particularly rare. Nonetheless, this case suggests that a regimen combining gemcitabine, paclitaxel, bevacizumab, and pembrolizumab may be a manageable therapeutic option for USRCS. Further research is warranted to elucidate the biological behavior of this malignancy and to validate the efficacy of such combination therapies in prospective studies.

## Data Availability

The original contributions presented in the study are included in the article/**Supplementary Material**. Further inquiries can be directed to the corresponding author/s.
